# *Sharia* (Islamic Law) Perspectives of COVID-19 Vaccines

**DOI:** 10.3389/fitd.2021.788188

**Published:** 2021-12-19

**Authors:** Yan Mardian, Kathryn Shaw-Shaliba, Muhammad Karyana, Chuen-Yen Lau

**Affiliations:** 1 Indonesia Research Partnership on Infectious Disease (INA-RESPOND), Jakarta, Indonesia; 2 National Institute of Allergy and Infectious Diseases, National Institutes of Health, Bethesda, MD, United States; 3 National Institute of Health Research and Development, Ministry of Health, Republic of Indonesia, Jakarta, Indonesia; 4 HIV Dynamics and Replication Program, Center for Cancer Research, National Cancer Institute, National Institutes of Health, Bethesda, MD, United States

**Keywords:** Halal certificate, *Sharia* (Islamic law), Fatwa, COVID-19 vaccines, islamic

## Abstract

The Coronavirus disease 2019 (COVID-19) pandemic has caused health, economic, and social challenges globally. Under these circumstances, effective vaccines play a critical role in saving lives, improving population health, and facilitating economic recovery. In Muslim-majority countries, Islamic jurisprudence, which places great importance on sanctity and safety of human life and protection of livelihoods, may influence vaccine uptake. Efforts to protect humans, such as vaccines, are highly encouraged in Islam. However, concerns about vaccine products’ *Halal* (permissible to consume by Islamic law) status and potential harm can inhibit acceptance. Fatwa councils agree that vaccines are necessary in the context of our current pandemic; receiving a COVID-19 vaccination is actually a form of compliance with Sharia law. Broader use of animal component free reagents during manufacturing may further increase acceptance among Muslims. We herein explain the interplay between *Sharia* (Islamic law) and scientific considerations in addressing the challenge of COVID-19 vaccine acceptance, particularly in Muslim populations.

## INTRODUCTION

COVID-19, caused by the severe acute respiratory syndrome coronavirus-2 (SARS-CoV-2) virus, ignited a pandemic in 2020 and continues to circulate. Elevations in case numbers, circulation of variants of concern and potential variants of high consequence have alarmed the world. A coordinated global response led to development of COVID-19 vaccines in record time. Major vaccination efforts are now ongoing throughout the world (1). Unfortunately, uptake has been hindered for several reasons, including religious beliefs (2). This article will provide an Islamic perspective on COVID-19 vaccines.

In Islam, every aspect of life should align with *Sharia* (Islamic law), or God’s will for humankind. The sources of Sharia are the *Al-Quran* (Islamic holy book) and *Al-Hadith* (record of the words, actions, and the silent approval of the Islamic Prophet Muhammad) (3, 4). To effectuate God’s will, Islamic scholars provide their interpretation through the Islamic body of law called *Fiqh* (Islamic jurisprudence). While *Sharia* is the decree of God, *Fiqh* is accomplished *via* analysis by *Ulama* (clerics) of *Al-Quran* and *Al-Hadith*. *Fiqh* is neither sacred nor fixed, as it results from human opinion at a certain place and time and can be modified according to circumstances (3). When Muslims need clarity, *Ulama* perform *ijtihad* (best efforts) based on their understanding of *Sharia* and issue a *Fatwa* (ruling) to address questions. Since *Fatwas* are based on *Fiqh* and *Ulama*s’ *ijtihad*, varying scientific background and religious experience of *Ulamas* or authorized institutions may engender multiple different rulings on an issue, including vaccines (5).

*Ulamas* formulate *Fatwas* regarding COVID-19 vaccines by considering *Sharia* sources and scientific studies. During the *Fatwa* formulation process, *Ulamas* attempt to weigh religious and scientific values fairly (6). Fatwas are of critical importance to vaccine acceptance in Muslim populations, which have long had concerns about purity of contents (7). We herein describe current COVID-19 vaccine ingredients and how various authorized Islamic regulators have formed the relevant *Fatwa*s. Understanding vaccine regulation under Islamic law provides insights for increasing vaccine acceptance in Muslim-majority countries, especially during a pandemic.

## VACCINE INGREDIENTS

Vaccines can be generally classified as live or non-live, which distinguishes those containing attenuated replicating strains of the relevant pathogen from those containing only pathogen components or killed whole organisms. In addition to the ‘traditional’ live and non-live vaccines, other platforms have been developed over the past few decades, including viral vectors and nucleic acid-based RNA vaccines (8). Several COVID-19 vaccines have been developed using these new technologies (9, 10). Vaccines contain essential or active components that induce an immune response conferring protection upon subsequent exposure to the target pathogen. Apart from these active components, the main ingredient is typically water. Other ingredients may be added, including adjuvants to improve immunogenicity, preservatives, emulsifiers (such as polysorbate 80), or stabilizers (e.g., gelatin or sorbitol) (11). These added ingredients are typically present in very small quantities.

Products used during manufacturing could also theoretically remain in the final product and are included as potential trace vaccine components (8). For example, inactivation with formaldehyde is commonly used to produce human and animal vaccines such as those against polio, hepatitis A, enterovirus 71, and influenza viruses (12). The formaldehyde is diluted to trace levels in the final product and does not pose a safety concern (13). Other components may include antibiotics, egg or yeast proteins, latex, glutaraldehyde, and acidity regulators (such as potassium or sodium salts). Except in the case of allergy, such as yellow fever vaccination in the context of true egg allergy, there is no evidence of risk to human health from these trace components (8).

COVID-19 vaccines use several different platforms, with varying active ingredients and excipients. Additionally, some approaches, such as mRNA-based vaccines, are synthetically created in a lab and therefore do not require cell culture during production (9). Composition of COVID-19 vaccines included in WHO’s Emergency Use Listing (EUL) is described in Table 1 (14–19).

## *HALAL* ASPECT OF VACCINES

Vaccination coverage varies with access, affordability, awareness, and acceptance. Vaccine acceptance is a crucial component of disease prevention, as vaccines are only effective if used. However, some Muslim individuals have concerns that vaccines and other pharmaceuticals may not be *Halal*, and are therefore more likely to remain unvaccinated (20). There is also heterogeneity in the influence of religion on vaccination practices amongst Muslim-majority countries. In Saudi Arabia, an Islamic theocracy, a survey showed that parents were highly confident in vaccines; even vaccine-hesitant parents did not view religion as prohibiting vaccination. Conversely in Pakistan, which has the world’s second-largest Muslim population after Indonesia, local rumours with religious undertones falsely asserted that the polio vaccine causes sterilization and contains porcine products. Pakistan continues to see polio outbreaks (21).

*Halal* products are those which are permitted according to the *Sharia* law. Typically, *Halal* refers to the permissibility to eat, drink, or act based on Islamic law and principles. Substances used in vaccine manufacturing may be of animal origin, including swine or derivatives, dead animals, or blood, which are *Haram* or forbidden for Muslims to consume (6, 22). In Islam, Muslims are required to follow *Sharia* law, which is authoritative. The Holy *Al-Quran* states: “*Therefore, (O Believers) eat of the lawful and good things that Allah has provided for you, and be grateful for His favours if it is true that you only worship Him. Indeed, Allah has forbidden you that which dies of itself, blood, and the flesh of swine; also, any flesh consecrated to something other than in the name of Allah. But whoever is compelled by necessity (to eat any of this) not intending to sin or transgress (regarding the quantity eaten), will find Allah Most Forgiving, Most Merciful*”. (Q.S. An-Nahl 16:114–115). The passage explains why Muslims abstain from using *Haram* material or consuming porcine products and derivatives (6, 23).

Swine are amongst the animals declared as *Haram* by *Sharia* law. Using their parts and derivatives in pharmaceuticals will render them non-permissible for consumption by Muslims (22). However, swine derivatives are commonly used in vaccine production, including porcine trypsin and porcine gelatine. Porcine trypsin extracted from the swine pancreas is a reagent used during the propagation stage of production of certain vaccines, e.g., inactivated polio and Japanese encephalitis virus vaccines, to remove or detach cells from the culture tank or vessels before harvesting. It may also be used during the final culture stage of virus production for vaccine activation, such as with influenza virus and rotavirus. Although semi-synthetic (recombinant) trypsin is commercially available, porcine trypsin is commonly used for its lower cost and availability. Porcine trypsin is washed from harvested cells before further processing. Its presence is typically assessed by validated techniques, studies of which have mostly demonstrated undetectable amounts of porcine trypsin in final products (22, 24).

Hydrolyzed porcine gelatine is a mixture of peptides and proteins produced by partial hydrolysis of collagen, typically extracted from swine skin, tendons, ligaments, bones, cartilage, or other components. Porcine gelatine is used in vaccines to stabilize and preserve active ingredients during freeze-drying and storage. Unlike food grade gelatine, the gelatine used for vaccine production is highly purified and broken down into peptides. Although only present in small amounts, a label stating “Contains trace quantities of porcine content” is sometimes required by local product registration policy (2, 22, 24).

## *FATWA* FORMULATION FOR COVID-19 VACCINES

In Muslim-populated countries, *Halal* certification administrators use the Holy *Al-Quran* as a guide for granting the *Halal* certificate to applicants. Administrators evaluate the cleanliness of the applicant’s premises and equipment, selection of ingredients, and cross-contamination between *Halal* and *non-Halal* products (25). “*Halal* pharmaceuticals” must contain only ingredients permitted by *Sharia* law. They must specifically: (1) be free of parts or derivatives of animals declared non-Halal by *Sharia* law or not slaughtered according to *Sharia* law; (2) not contain *najs* (impurities); and (3) not be poisonous, intoxicating, or pose a health hazard to users when taken according to prescription (22). However, interpretation and implementation of *Halal* pharmaceutical certification varies between countries.

Indonesia has the world’s largest Muslim population, with 87% of its 277 million inhabitants identifying as Muslim. It is diverse in terms of language, ethnicity, and cultural background which, in addition to religion, impact vaccine perception. A *fatwa*, or ruling under Islamic law, to declare *Halal* status of a vaccine can be issued by Indonesia’s authorized *Halal* certification administrators, Indonesian Ulama Council (MUI) (21). The first COVID-19 vaccine authorized in the country, Sinovac, was granted a *Halal* and holy certificate by the MUI on January 11, 2021. The certification states that this vaccine does not use porcine trypsin or other animal enzymes during manufacturing (26). The MUI subsequently issued a fatwa on March 19, 2021 stating that the AstraZeneca COVID-19 vaccine is “*Haram-permittable*”. MUI claimed it is *Haram* because it uses porcine trypsin during the early production process, but is *permittable* to use (or *Mubah*) due to the urgency of addressing COVID-19 (27). Notably, several other nations with Muslim majority populations, including Saudi Arabia, the United Arab Emirates, Egypt, and Malaysia, use AstraZeneca vaccine without concern over whether it is *Halal* or *Haram* (28). Additionally, the Indonesian Food and Drug Monitoring Agency (BPOM) and WHO have confirmed the absence of porcine products in the AstraZeneca vaccine (16, 28). Some COVID-19 vaccines, the Sinopharm and Pfizer-BioNTech vaccines are also deemed *Haram-permittable*, and can be used for emergencies. Other COVID-19 vaccines that have been granted EUA from BPOM, such as Moderna, J&J, Sputnik V, and CanSino, are not yet *Halal/Haram* certified by MUI as of September 2021 (https://mui.or.id/).

Some fear that COVID-19 vaccines will suffer the same fate as the measles−rubella combination vaccine introduced to Indonesia in 2017 (28). At that time, MUI issued a *fatwa* that the vaccine was *Haram* due to porcine components being used in the manufacturing process. After the MUI *fatwa* declaring it *Haram*, uptake declined precipitously. While all six provinces on Java reached the 95% coverage target and saw measles and rubella cases decline by over 90%, coverage of children on other islands has reached only 68%. In Aceh, the only province allowed to practice *Sharia* law, coverage is only 8%, putting Indonesia at risk for a measles outbreak (21, 29). COVID-19 vaccine coverage disparities are also seen amongst provinces. Aceh again demonstrates extremely low coverage, with only 11.8% of its target population being fully vaccinated (https://vaksin.kemkes.go.id/). Religious considerations should be addressed in vaccine roll-out, with the engagement of religious leaders as a priority, since the *fatwa* specifically permits the use of non-*Halal* vaccines in the emergency (21).

## RECONCILING *SHARIA* LAW WITH CONTEMPORARY NEEDS

Despite potential conflict with *Sharia* law, clerics in several Muslim countries have accepted vaccines that utilize impure substances such as porcine gelatine in their production process. They concluded that gelatine in vaccines is *Halal* because it has undergone hydrolysis, which purifies it under an Islamic legal concept called *istihalah* (perfect change) (2, 30). *Istihalah* refers to alteration of physicochemical nature to change a non-acceptable *Haram* product to an acceptable *Halal* form (31). This view is based on the principle of “transformation” in *Sharia* law, which is applied to vinegar production from wine (2). Vaccines become acceptable if the impure component is completely transformed into a new substance, different from its origin. Transformation of impure substances through downstream processing, e.g. filtration, to render them negligible in the final product is similarly used with other pharmaceuticals such as Heparin (porcine enzymes) and the Rotavirus vaccine (porcine trypsin). In Muslim jurisprudence, these processes accomplish *istihalah* and render the final product permissible for Muslim use (2, 32, 33).

In addition to *istihalah*, *istihlak* (mixing) can convert an unclean substance into one that is clean (34). ‘*Istihlak*’ refers to mixing of a substance with another until it is dissolved, causing loss of properties even though the substance still exists. Thus an unclean product can be mixed with a more dominant clean product to vanquish the unclean characteristics. This concept is adapted from the *Hadith*, which explains the characteristic of two *kolah* (about 216–270 litres) of water: “*The Prophet was asked about the status of stagnant water being licked by reptiles and wild animals (i.e., whether the water is still clean). Then the Prophet said,* “*If the water exceeds two kolah, then it does not become unclean*”. Another hadith explains “*If the water exceeds two kolah, and is then mixed with the unclean, it does not become unclean as long as there is no change in its smell and taste*” (33).

The concepts of *istihalah* and *istihlak* are not universally accepted by all *Ulamas* and authorization councils which determine *Halal* status. Acceptance by *fatwa* institutions varies based upon interpretation of Sharia law (33). However, during the COVID-19 pandemic, all councils agreed that an effective and safe COVID vaccine is a basic necessity, or *darurat* (emergency). Recognition as *darurat* justifies consumption of *Haram* product if needed in an emergency situation (35). The Holy *al-Quran* states: “*He has only forbidden you what dies of itself (carrion) and blood and the* *flesh of swine, and that which is slaughtered as a sacrifice for others than Allah. But if one is forced by necessity without willful disobedience nor transgressing due limits, then there is no sin on them. Truly, Allah is Oft-Forgiving, Most Merciful*” (Q.S. Al-Baqarah 2:173). COVID-19 vaccines are recognized as necessary or critical for saving lives and ensuring that societies can function. They are equivalent in status to other established basic human needs such as food and shelter, and therefore are eligible to be classified as *darurat*. A vaccine that protects against harm from SARS-CoV-2 is essential to uphold the principles of sanctity of human life and avoidance of harm (2).

The COVID-19 vaccine development process is also consistent with the principle of the avoidance of harm in Islamic jurisprudence. All vaccines approved for public use undergo stringent safety and efficacy evaluation, which conform to requirements of national ethics bodies (36). Vaccine development reflects the *Qur*’*an*’s concept of prevention, or *wiqaya*, which can refer to preventive actions such as against hell-fire, punishment, greed, bad acts, harm and heat. The *Qur*’*an* concludes that prevention is one of the laws of God, so it also applies to the role of vaccination for preventing harm to humans (2, 37). This consideration in combination with scientific data provided a basis for *fatwa* councils worldwide to publish Islamic legal rulings permitting use of recently developed COVID-19 vaccines, listed in Table 2.

Vaccines are required to be manufactured under current good manufacturing practices (cGMP) (38–40). cGMP requires that products for human administration should not be contaminated with extraneous materials, including those of animal origin (9 C.F.R. § 113 and 21 C.F.R. 610) (38, 41, 42). Regulations addressing cell culture based biologics became particularly important after the discovery that variant Creutzfeldt-Jakob disease (vCJD) could be transmitted between species by “prions” or infectious protein, without involving any nucleic acids (43). There is a current push to utilize animal-component-free (ACF) or xeno-free reagents (44). In recent years, progress has been made to generate serum-free media alternatives and ACF products for pharmaceutical manufacturing, including vaccine production. As growth media influences cells’ characteristics, safety and efficacy, comparative studies are essential to understanding the differences, advantages, and challenges associated with specific serum-free/ACF formulations. Nevertheless, the use of ACF reagents during vaccine manufacturing aligns with the *Halal* way in *Sharia*, without depending on the acceptance of *Istihalah* and *Istihlak* concepts. Thus, the broader use of ACF products in vaccine manufacturing, especially in Muslim countries, could ameliorate vaccine hesitancy associated with Muslim religious beliefs.

## CONCLUSIONS

From the Islamic point of view, preserving life is aligned with preserving religion (35). Muslims who refuse to receive COVID-19 vaccines may be regarded as acting against *Sharia* law. Yet *Halal* certification is only one of many issues that may affect vaccine uptake. The anti-vaccination movement, concerns about long term side effects, accessibility and mis-information pose additional challenges. Effective scientific discourse and communication, including regular engagement with Islamic law scholars, *Ulamas*, and national regulatory agencies, will be critical for achieving vaccination targets (45).

Individual decisions about accepting COVID-19 vaccines are multifactorial. The *Halal* issue may pose a significant challenge amongst Muslim populations. *Fatwa* councils worldwide have used both *sharia* and scientific approaches to grant *Halal* certificates for COVID-19 vaccines. Yet there have been inconsistencies across regions. For example, the AstraZeneca COVID-19 vaccine is considered *Haram* by the Indonesian council but *Halal* by other councils. Nonetheless, all *fatwa* councils agree that vaccines are necessary in the context of our current pandemic, and thus receiving a COVID-19 vaccination is actually a form of compliance with *Sharia* law. Broader use of ACF reagents during manufacturing may further increase acceptance among Muslims.

## Figures and Tables

**TABLE 1 |:** Active and excipient ingredients of COVID-19 vaccines included in WHO’s Emergency Use Listing (EUL).

COVID-19 Vaccine (developer)	Platform	Active Ingredients	Other Ingredients	Cell-line used for vaccine manufacture	Animal or human origin excipients in final product
Sinovac-Coronavac (Sinovac)	Inactivated	Each 0.5 mL dose is composed of 3 μg of β-propiolactone-inactivated SARS-CoV-2 virus (Wuhan/WIV04/2019 strain)	Excipients are aluminium hydroxide adjuvant, disodium hydrogen phosphate, sodium dihydrogen phosphate, sodium chloride, and water for injection. The vaccine does not contain preservatives.	Vero cell, extracted from an African green monkey	None
BIBP (China National Biotec Group (CNBG), Sinopharm)	Inactivated	Each 0.5 mL dose is composed of 6.5 U (4 μg) of β-propiolactone-inactivated SARS-CoV-2 antigens [19nCOV-CDC-TAN-HB02 strain (HB02 strain)]	Excipients are aluminium hydroxide adjuvant and phosphate-buffered saline (PBS), which composed of disodium hydrogen phosphate dodecahydrate, sodium dihydrogen phosphate, and sodium chloride.	Vero cell, extracted from an African green monkey	None
Vaxzevria or AstraZeneca/ AZD1222 vaccine (Oxford University and AstraZeneca)	Viral Vector	Each 0.5 mL dose contains 5 × 10^10^ ChAdOx1-S (recombinant) viral particles, composed of a replication-deficient chimpanzee adenovirus vector encoding the S glycoprotein of SARS-CoV-2	Excipients are L-histidine, L-histidine hydrochloride monohydrate, magnesium chloride hexahydrate, polysorbate 80, ethanol, sucrose, sodium chloride, disodium edetate dihydrate and water for injection.	T-REx-293 cells, a derivative of the human embryonic kidney (HEK 293) cell line, descended from tissue taken from a 1973 abortion in the Netherlands from an undisclosed source.	None
Janssen/Ad26.COV 2.S (Johnson & Johnson)	Viral Vector	Each 0.5 ml dose contains 5 × 10^10^ AD26.COV2.S viral particles, composed of replication-incompetent adenovirus type 26 (Ad26)-vectored monovalent vaccine encoding the SARS-CoV-2 spike (S) protein from the Wuhan- Hu-1 isolate (GenBank accession number MN908947), stabilized in its prefusion conformation	Excipients are citric acid monohydrate, trisodium citrate dihydrate, ethanol, 2-hydroxypropyl-beta-cyclodextrin (HBCD), polysorbate 80, sodium chloride, sodium hydroxide, and hydrochloric acid. The vaccine does not contain preservatives.	PER.C6G TetR cell line, derived from human embryonic retinal cells, obtained in 1985 from fetal retina tissue	None
Comirnaty or BNT162b2 (Pfizer-BioNTech)	mRNA	Each 30 mcg/0.3mL dose contains a highly purified single-stranded, 5’-capped mRNA, encoding a P2 mutant spike protein (PS 2) of SARS-CoV-2 virus.	Excipients are ALC-0315, ALC-0159 (polyethylene glycol), cholesterol, potassium chloride, potassium dihydrogen phosphate, sodium chloride, disodium hydrogen phosphate dihydrate, sucrose, and water for injection.	None (cell-free *in vitro* transcription process from DNA templates)	None
Spikevax or mRNA-1273 vaccine (Moderna)	mRNA	Each 100 mcg/0.5 mL dose contains a synthetic mRNA (single-stranded, 5’-capped) encoding the prefusion-stabilized spike glycoprotein (S) of SARS-CoV-2 virus.	Excipients are lipids [SM-102, 1,2-dimyristoyl-rac-glycero-3-methoxypolyethylene glycol-2000 (PEG2000-DMG), cholesterol, and 1,2-distearoyl-sn-glycero-3-phosphocholine (DSPC)], tromethamine, tromethamine hydrochloride, acetic acid, sodium acetate, and sucrose.	None (cell-free *in vitro* transcription process from DNA templates)	None

**TABLE 2 |:** *Fatwa* (Ruling) status and considerations for granting *Halal* certification of COVID-19 vaccines in several countries.

*Halal* certification administrators	Considerations for *Halal* Certification for COVID-19 Vaccines	Conclusion Fatwa (Islamic Ruling)
Ulama Council of Indonesia (MUI) (www.mui.or.id)	Vaccines are required to use *Halal* (permissible) and holy (clean) ingredients. In granting the fatwa for vaccines, three principles must be met:	Sinovac-Coronavac is deemed “holy and *Halal*” AstraZeneca, Sinopharm and Pfizer-BioNTech vaccine are deemed *Maram* since they use porcine trypsin during manufacturing or contain animal products. Vaccination is permissible by Islamic laws because there is an urgent need to vaccinate immediately and sufficient clean and *Halal* product is not available
	Raw materials, additives, and auxiliary materials must be *Halal* The manufacturing process must be guaranteed not to be contaminated with impurities *(najs)* The manufacturer has a system to guarantee *Halal* processes throughout production. However, immunization with non-*Halal* vaccines may be allowed under certain conditions: When used in an emergency condition, known as *al-hajat,* or *Halal* and holy vaccine ingredients have not been found and there is information from competent and trusted medical personnel that no *Halal* vaccine is available. If non-immunization will cause death, serious illness, or permanent life-threatening disability, then legal immunization is mandatory.	Note: This fatwa is questionable since MUI’s claim that trypsin of a porcine origin was used in the manufacturing process differs from the vaccines manufacturers’ statement that declares NO animal or human origin excipients present in their final products.
Fatwa Council of the United Arab Emirates (www.awqaf.gov.ae)	COVID-19 is a highly contagious disease that could cause death or permanent damage to humans. The vaccine is the only way to protect against this virus. COVID-19 vaccines are in compliance with Sharia (Islamic law) objectives to protect the human body and with other relevant Islamic rulings. Even though the vaccine might contain non-*Halal* ingredients, it is acceptable to use it when there are no alternatives. Vaccines are categorized as preventive medicines that help society avoid the high risk of infection.	COVID-19 vaccines are *Halal*
Fiqh Academy of the Organization of Islamic Cooperation, Saudi Arabia (www.iifa-aifi.org)	Specialists in pharmacology and preventive medicine confirm that the COVID-19 vaccines consist of the messenger RNA (ribonucleic acid) of SARS-CoV-2, recombinant DNA, the common cold virus (adenovirus), other viruses, bacteria, compounds of the Quillaja tree and plants, and auxiliary substances such as potassium, sodium, magnesium, phosphate, acetic acid, tromethamine hydrochloride, diacetate, polysorbate, sucrose, lipids, cholesterol, histidine, ethanol and water. These vaccines do not contain swine or human derivatives During production, chemical reactions and transformations occur, which fall within the *Sharia* rulings regarding metamorphosis in Islamic jurisprudence (*istihalah*). Vaccination becomes mandatory if the government obligates it, because the government’s rulings uphold public interests	COVID-19 Vaccines are permissible according to Sharia law (*Halal*).
Islamic Religious Council of Singapore (www.muis.gov.sg)	Vaccines are a basic necessity Vaccines are safe and efficacious Ingredients used in vaccines are permissible.	Thus far, COVID-19 vaccines (Pfizer-BioNTech and Moderna) in the national program do not diverge from *Sharia* considerations. As such, we hold the position that a COVID-19 vaccine is permissible for Muslim use (*Halal*).
Taiwan *Halal* Certification Bodies (Chinese Muslim Association and Taipei Grand Mosque) (www.cdc.gov.tw)	None of the COVID-19 vaccines granted Emergency Use Authorization (EUA) in Taiwan, including the vaccines from AstraZeneca, Moderna, Pfizer-BioNTech and Medigen, contain swine cells Many large fatwa councils around the world have already published Islamic legal rulings regarding the permissibility of the recently developed COVID-19 vaccines.	COVID-19 vaccines are *Halal* as they do not contain animal products of any kind.
The Islamic Food and Nutrition Council of America (IFANCA) or Assembly of Muslim Jourist of America (AMJA) (www.amjaonline.org)	Vaccine approval is not a decision made by one person or company, but by the Food and Drug Administration (FDA) and similar agencies in other countries which follow stringent practices that consider the risk-benefit ratio, congruent with the principles of Sharia As for the possibility of cultivating some viruses in porcine cells (if actually taking place), the vaccine would usually contain none of the cellular parts. This makes the *najasah* (impurity) occur only by proximity (*mujawarah*). In addition to being microscopic, trivial things of this nature should be excused, particularly when the matter pertains to protecting people from this disastrous pandemic. Medical experts in virology and vaccines who are involved in COVID-19 research have clearly stated that the benefits of COVID-19 vaccinations far outweigh their risks.	All the vaccines approved in the US (Pfizer-BioNTech, Moderna, J&J) have been deemed permissible to use (*Halal*).
Wifaqul Ulama (Britain) (www.wifaqululama.co.uk)	Considerations for the AstraZeneca Vaccine: Ethanol content is very small (0.002 g of alcohol/ethanol per dose of 0.5 mL). This is not enough to cause any noticeable effects. Vaccine production uses the HEK 293 TREX producer cell line originally derived from kidney cells from a female foetus aborted in the 1970s. None of the original foetus derived cells are used in the vaccine and purification was performed by CsCl gradient ultracentrifugation to remove the cell culture material. Considerations for the Pfizer-BioNTech Vaccine: The main ingredient in this vaccine is nucleoside-modified messenger RNA and it is not sourced from animals All excipients apart from cholesterol are chemically synthesised. The following official statement was released: “The Medicines and Healthcare products Agency confirms that the Pfizer/BioNTech COVID-19 vaccine does not contain any components of animal origin.” Considerations for the Moderna Vaccine: The following official statement was released, “The Medicines and Healthcare products Agency can confirm that the Moderna COVID-19 vaccine does not contain any components of animal origin.”	In the light of the available data, official statement and clarification, Wifaqul Ulama concluded that COVID-19 vaccines (AstraZeneca, Pfizer, and Moderna) are permissible for British Muslims (*Halal*).
Australian Fatwa Council (www.anic.org.au)	COVID-19 Vaccines are safe In the final vaccine product, there are no gelatine, animal products or cells of any kind remaining None of the vaccine types appears to affect the DNA inside our cells, nor have any vaccines (which have been used for decades) been shown to affect human DNA Medical experts in virology and vaccines who are involved in COVID-19 research have clearly stated that the benefits of these vaccinations far outweigh their risks.	The COVID-19 Vaccines (Pfizer-BioNTech, Moderna, AstraZeneca) are permissible according to Islamic law as there is no known religious harm attributed to being vaccinated nor does it contain any forbidden substances (*Halal*).

## Data Availability

The datasets presented in this article are not readily available because the study did not analyze any particular data, we reviewed published articles and Islamic laws. Requests to access the datasets should be directed to ymardian@ina-respond.net.

## References

[1] Ball P The Lightning-Fast Quest for COVID Vaccines - and What It Means for Other Diseases. Nature (2021) 589(7840):16–8. doi: 10.1038/d41586-020-03626-1 33340018

[2] Grabenstein JD. What the World’s Religions Teach, Applied to Vaccines and Immune Globulins. Vaccine (2013) 31(16):2011–23. doi: 10.1016/j.vaccine.2013.02.026 23499565

[3] Yusroh Y, Rahman MZA. Mapping Contemporary Islamic Jurisprudence of Muḥammad Saʻīd Al-’Ashmāwī and Muḥammad Shaḥrūr. IJISH (Int J Islamic Stud Humanit) (2018) 1(1):32–46. doi: 10.26555/ijish.v1i1.132

[4] Thalib P Distinction of Characteristics Sharia and Fiqh on Islamic Law. Yuridika (2018) 33(3):439–52. doi: 10.20473/ydk.v33i3.9459

[5] Khoiri N The Mapping of Renewal of ‘Usul Fiqh’thoughts in Indonesia. Int J Language Res Educ Stud (2017) 1(1):18–33. doi: 10.30575/2017081202

[6] Ab Latiff J, Zakaria Z. The Challenges in Implementation of Halal Vaccine Certification in Malaysia. J Food Pharm Sci (2021) 9(1):2. doi: 10.22146/jfps.1147

[7] Wong LP, Wong PF, AbuBakar S. Vaccine Hesitancy and the Resurgence of Vaccine Preventable Diseases: The Way Forward for Malaysia, a Southeast Asian Country. Hum Vaccines Immunother (2020) 16(7):1511–20. doi: 10.1080/21645515.2019.1706935 PMC7482793 31977285

[8] Pollard AJ, Bijker EM. A Guide to Vaccinology: From Basic Principles to New Developments. Nat Rev Immunol (2021) 21(2):83–100. doi: 10.1038/s41577-020-00479-7 33353987 PMC7754704

[9] Mathew S, Faheem M, Hassain NA, Benslimane FM, Thani AAA, Zaraket H, Platforms Exploited for SARS-Cov-2 Vaccine Development. Vaccines (Basel) (2020) 9(1):11. doi: 10.3390/vaccines9010011 33375677 PMC7824029

[10] Li Y, Tenchov R, Smoot J, Liu C, Watkins S, Zhou Q. A Comprehensive Review of the Global Efforts on COVID-19 Vaccine Development. ACS Cent Sci (2021) 7(4):512–33. doi: 10.1021/acscentsci.1c00120 34056083 PMC8029445

[11] Kocourkova A, Honegr J, Kuca K, Danova J. Vaccine Ingredients: Components That Influence Vaccine Efficacy. Mini Rev Med Chem (2017) 17(5):451–66. doi: 10.2174/1389557516666160801103303 27488583

[12] Wilton T, Dunn G, Eastwood D, Minor PD, Martin J. Effect of Formaldehyde Inactivation on Poliovirus. J Virol (2014) 88(20):11955–64. doi: 10.1128/JVI.01809-14 25100844 PMC4178759

[13] U. S. Food and Drug Administration. Common Ingredients in US Licensed Vaccines (2014). Available at: https://www.fda.gov/vaccines-blood-biologics/safety-availability-biologics/common-ingredients-us-licensed-vaccines.

[14] World Health Organization. Background Document on the Inactivated Vaccine Sinovac-Coronavac Against COVID-19. Geneva, Switzerland: World Health Organization (2021). Available at: https://www.who.int/publications/i/item/WHO-2019-nCoV-vaccines-SAGE_recommendation-Sinovac-CoronaVac-background-2021.1.

[15] World Health Organization. Background Document on the Inactivated COVID-19 Vaccine BIBP Developed by China National Biotec Group (CNBG). Geneva, Switzerland: World Health Organization Rome, Italy (2021). Available at: https://www.who.int/publications/i/item/WHO-2019-nCoV-vaccines-SAGE_recommendation-BIBP-background-2021.1.

[16] World Health Organization. Background Document on the AZD1222 Vaccine Against COVID-19 Developed by Oxford University and Astrazeneca: Background Document to the WHO Interim Recommendations for Use of the AZD1222 (Chadox1-s [Recombinant]) Vaccine Against COVID19 Developed by Oxford. Geneva, Switzerland: World Health Organization (2021). Available at: https://www.who.int/publications/i/item/background-document-on-the-azd1222-vaccine-against-covid-19-developed-by-oxford-university-and-astrazeneca.

[17] World Health Organization. Interim Recommendations for the Use of the Janssen Ad26. COV2. s (COVID-19) Vaccine. Background Document on the Janssen Ad26COV2S (COVID-19) Vaccine: Background Document to the WHO Interim Recommendations for Use of Ad26COV2S (COVID-19) Vaccine (2021). Available at: https://www.who.int/publications/i/item/WHO-2019-nCoV-vaccines-SAGE-recommendation-Ad26.COV2.S-background-2021.1.

[18] World Health Organization. Background Document on Mrna Vaccine BNT162b2 (Pfizer-Biontech) Against COVID-19. License: CC by-NC-SA 3.0 IGO (2021). Available at: https://www.who.int/publications/i/item/background-document-on-mrna-vaccine-bnt162b2-(pfizer-biontech)-against-covid-19.

[19] World Health Organization. Background Document on the Mrna-1273 Vaccine (Moderna) Against COVID-19: Background Document to the WHO Interim Recommendations for Use of the Mrna-1273 Vaccine (Moderna). Geneva, Switzerland: World Health Organization (2021). Available at: https://www.who.int/publications/i/item/background-document-on-the-mrna-1273-vaccine-(moderna)-against-covid-19.

[20] Kabir R, Mahmud I, Chowdhury MTH, Vinnakota D, Jahan SS, Siddika N, COVID-19 Vaccination Intent and Willingness to Pay in Bangladesh: A Cross-Sectional Study. Vaccines (Basel) (2021) 9(5):416. doi: 10.3390/vaccines9050416 33919254 PMC8143282

[21] Harapan H, Shields N, Kachoria AG, Shotwell A, Wagner AL. Religion and Measles Vaccination in Indonesia, 1991–2017. Am J Prev Med (2021) 60(1 Suppl 1):S44–52. doi: 10.1016/j.amepre.2020.07.029 33189503 PMC7894975

[22] Khoo YSK, Ghani AA, Navamukundan AA, Jahis R, Gamil A. Unique Product Quality Considerations in Vaccine Development, Registration and New Program Implementation in Malaysia. Hum Vaccines Immunother (2020) 16(3):530–8. doi: 10.1080/21645515.2019.1667206 PMC7227723 31652090

[23] Al-Teinaz YR. What is Halal Food? In: The Halal Food Handbook (2020). Hoboken, New Jersey: Wiley Online Library. p. 7–26.

[24] Agency EM. Guideline on the Use of Porcine Trypsin Used in the Manufacture of Human Biological Medicinal Products (2013). Available at: http://www.ema.europa.eu/docs/en_GB/document_library/Scientific_guideline/2013/03/WC500139532.pdf.

[25] Latif IA, Mohamed Z, Sharifuddin J, Abdullah AM, Ismail MM. A Comparative Analysis of Global Halal Certification Requirements. J Food Prod Mark (2014) 20(sup1):85–101. doi: 10.1080/10454446.2014.921869

[26] Indonesian Ulema Council. Fatwa Majelis Ulama Indonesia Nomor : 02 Tahun 2021 Tentang Produk Vaksin Covid-19 Dari Sinovac Life Sciences, Co. Ltd China Dan PT Biofarma (2021). Available at: https://mui.or.id/produk/fatwa/29485/fatwa-mui-no-02-tahun-2021-tentang-produk-vaksin-covid-19-dari-sinovac-life-sciences-co-ltd-china-dan-pt-biofarma/.

[27] Indonesian Ulema Council. Fatwa MUI No 14 Tahun 2021 Tentang Hukum Penggunaan Vaksin Covid-19 Produk Astrazeneca (2021). Available at: https://mui.or.id/produk/fatwa/29883/fatwa-mui-hukum-penggunaan-vaksin-covid-19-produk-astrazeneca/.

[28] Tempo. We Need Science, Not Fatwas (2021). Available at: https://en.tempo.co/read/1446020/we-need-science-not-fatwas.

[29] Indonesian Rochmyaningsih D. ‘Vaccine Fatwa’sends Measles Immunization Rates Plummeting. Science Magazine (2018). Available at: https://www.science.org/news/2018/11/indonesian-vaccine-fatwa-sends-measles-immunization-rates-plummeting.

[30] World Health Organization RO for the EM. Statement Arising From a Seminar Held by the Islamic Organization for Medical Sciences on ‘The Judicially Prohibited and Impure Substances in Foodstuff and Drugs’ (2001). Available at: www.immunize.org/concerns/porcine.pdf.

[31] Jahangir M, Mehmood Z, Saifullah, Bashir Q, Mehboob F, Ali K. Halal Status of Ingredients After Physicochemical Alteration (Istihalah). Trends Food Sci Technol (2016) 47:78–81. doi: 10.1016/j.tifs.2015.10.011

[32] Rochmyaningsih D Indonesian Fatwa Causes Immunization Rates to Drop. Sci (New York NY) United States (2018) 362:628–9. doi: 10.1126/science.362.6415.628 30409867

[33] Rosman AS, Khan A, Fadzillah NA, Samat AB. Fatwa Debate on Porcine Derivatives in Vaccine From the Concept of Physical and Chemical Transformation (Istihalah) in Islamic Jurisprudence and Science. J Crit Rev (2020) 7(7):1037–45. doi: 10.31838/jcr.07.07.189

[34] Abubakar A, Hukum Vaksin MR. Teori Istihalah Dan Istihlak Versus Fatwa MUI. Media Syari’ah: Wahana Kajian Hukum Islam Dan Pranata Sosial (2021) 23(1):1–15. doi: 10.22373/jms.v23i1.8485

[35] Sholeh MAN, Helmi MI. The COVID-19 Vaccination: Realization on Halal Vaccines for Benefits. Samarah: J Hukum Keluarga Dan Hukum Islam (2021) 5(1):174–90. doi: 10.22373/sjhk.v5i1.9769

[36] Corey L, Mascola JR, Fauci AS, Collins FS. A Strategic Approach to COVID-19 Vaccine R&D. Sci (New York NY) (2020) 368(6494):948–50. doi: 10.1126/science.abc5312 32393526

[37] Kasule O Islamic Legal Guidelines on Polio Vaccination in India. 16th Session of the Fiqh Academy of India (2007). Available at: http://omarkasule-04.tripod.com/id1406.html.

[38] US FDA. Guidance for Industry: Characterization and Qualification of Cell Substrates and Other Biological Materials Used in the Production of Viral Vaccines for Infectious Disease Indications. In: Center for Biologics Evaluation and Research (2010). White Oak, Maryland: Food and Drug Administration. Available at: https://www.fda.gov/media/78428/download.

[39] US FDA. Facts About the Current Good Manufacturing Practices (Cgmps) (2015). Available at: https://www.fda.gov/drugs/pharmaceutical-quality-resources/facts-about-current-good-manufacturing-practices-cgmps.

[40] World Health Organization. Good Manufacturing Practices for Biological Products. In: Who Technical Report Series, vol. 822. (1992). p. 20–9. Geneva, Switzerland: World Health Organization. Available at: https://www.who.int/biologicals/publications/trs/areas/vaccines/gmp/WHO_TRS_822_A1.pdf?ua=1.

[41] Code US, Law P. 21 C.F.R. § 610. In: Code of Federal Regulations (2013). Washington, D.C: Office of the Federal Register. Available at: https://www.ecfr.gov/current/title-21/chapter-I/subchapter-F/part-610.

[42] Code US, Law P. 9 C.F.R. § 113. In: Code of Federal Regulations (2017). Washington, D.C: Office of the Federal Register. Available at: https://www.ecfr.gov/current/title-9/chapter-I/subchapter-E/part-113.

[43] Institute of Medicine (US) Forum on Microbial Threats. SAddressing Foodborne Threats to Health: Policies, Practices, and Global Coordination: Workshop SummaryS. In: Reporting Foodborne Threats: The Case of Bovine Sp. Washington (DC: National Academies Press (US (2006). Available at: https://www.ncbi.nlm.nih.gov/books/NBK57084/. National Academies Press (US). 21850788

[44] Fletcher T, Harris H. Safety Drives Innovation in Animal-Component-Free Cell-Culture Media Technology. In: Safety (2016). Iselin, New Jersey: BioPharm International. Available at: http://www.processdevelopmentforum.com/articles/safety-drives-innovation-in-animal-component-free-cell-culture-media-technology/.

[45] Rosenbaum L Escaping Catch-22 — Overcoming Covid Vaccine Hesitancy. New Engl J Med (2021) 384(14):1367–71. doi: 10.1056/NEJMms2101220 33577150

